# Neurogenesis in the adult brain functionally contributes to the maintenance of chronic neuropathic pain

**DOI:** 10.1038/s41598-021-97093-x

**Published:** 2021-09-17

**Authors:** Linette Liqi Tan, Julieta Alfonso, Hannah Monyer, Rohini Kuner

**Affiliations:** 1 Institute of Pharmacology, Heidelberg University, Im Neuenheimer Feld 366, 69120 Heidelberg, Germany; 2 Department of Clinical Neurobiology, University Hospital Heidelberg and German Cancer Research Center, Im Neuenheimer Feld 280, 69120 Heidelberg, Germany

**Keywords:** Neuroscience, Diseases of the nervous system, Chronic pain

## Abstract

Maladaptive adult neurogenesis in the mammalian brain has been associated with diverse behaviors including disrupted learning, negative mood disorders and psychiatric conditions. However, its functional role in the generation and maintenance of chronic pathological pain has not yet been elucidated. Using an inducible genetic deletion in vivo mouse model, different behavioural paradigms and home cage monitoring systems, we show that an absence of adult neurogenesis does not impact the development of neuropathic injury-induced peripheral nociceptive hypersensitivity, but rather promotes the recovery of pathological pain as well as improves parameters associated with the state of well-being of the injured mice. These results provide a mechanistic insight into the mechanisms of chronic pain and implicate neurogenic processes as a potential therapeutic target for reducing pain and improving the quality of life for patients.

## Introduction

Throughout life, neural stem cells in the adult mammalian brain are capable of initiating cell proliferation and neuronal differentiation. Over the past two decades, in particular, considerable research progress has highlighted the notion that new neurons generated in adulthood have crucial roles in healthy brain functions, including learning, mood and stress regulation and cognitive processes. Maladaptive neurogenic processes have also been proposed to be associated with aging and several neuropsychiatric and neurodegenerative disorders^1–3^.

Within the adult rodent brain, the subgranular zone of the dentate gyrus (DG) and the subventricular zone (SVZ) are the two best-studied neurogenic niches where adult neurogenesis processes are well-described. Additionally, recent evidence highlights the presence of novel neurogenic areas, including the striatum, cortex, amygdala and substantia nigra, where new neurons either arise from migrating progenitor cells originating in the SVZ or exist as local pools within these regions^1,4–7^, suggesting an extensive array of brain functions that these proliferating cells partake in daily life.

Recently, there has been a growing interest in the role of adult neurogenesis in chronic pain and its associated mental comorbidities including depression and disrupted learning^1^. For instance, mice with neuropathic pain display substantially reduced hippocampal neurogenesis and altered short-term synaptic plasticity that is partly due to disrupted tumor necrosis factor receptor 1 and brain-derived neurotrophic factor signalling^8–10^. Furthermore, the process of neurogenesis during pathological pain also appears to be highly susceptible to environmental factors such as stress from chronic immobilization, which exacerbated the loss of neuroblasts and reduced survival of new mature neurons in hippocampal granular layers^11^. Additionally, our recent work has also demonstrated that pathological pain enhances neurogenesis of SVZ-derived adult-born medium spiny neurons that populate the ventral striatum^12^. However, to date, it remains unclear if and how active adult neurogenesis becomes critical over the course of pathological pain. Understanding these processes is extremely relevant especially since the mechanisms underlying pain chronicity remains not fully understood and consequently, available therapeutic targets are not consistently effective for patients.

In this study, we employed a genetic approach that enabled an induced irreversible loss of both hippocampal and SVZ neurogenesis in mice specifically before the onset of injury, in order to investigate the role of adult neurogenesis at various stages of pain chronicity as well as its impact on the quality of life and mobility induced by peripheral neuropathy.

## Materials and methods

### Animals

**Figure 1:**
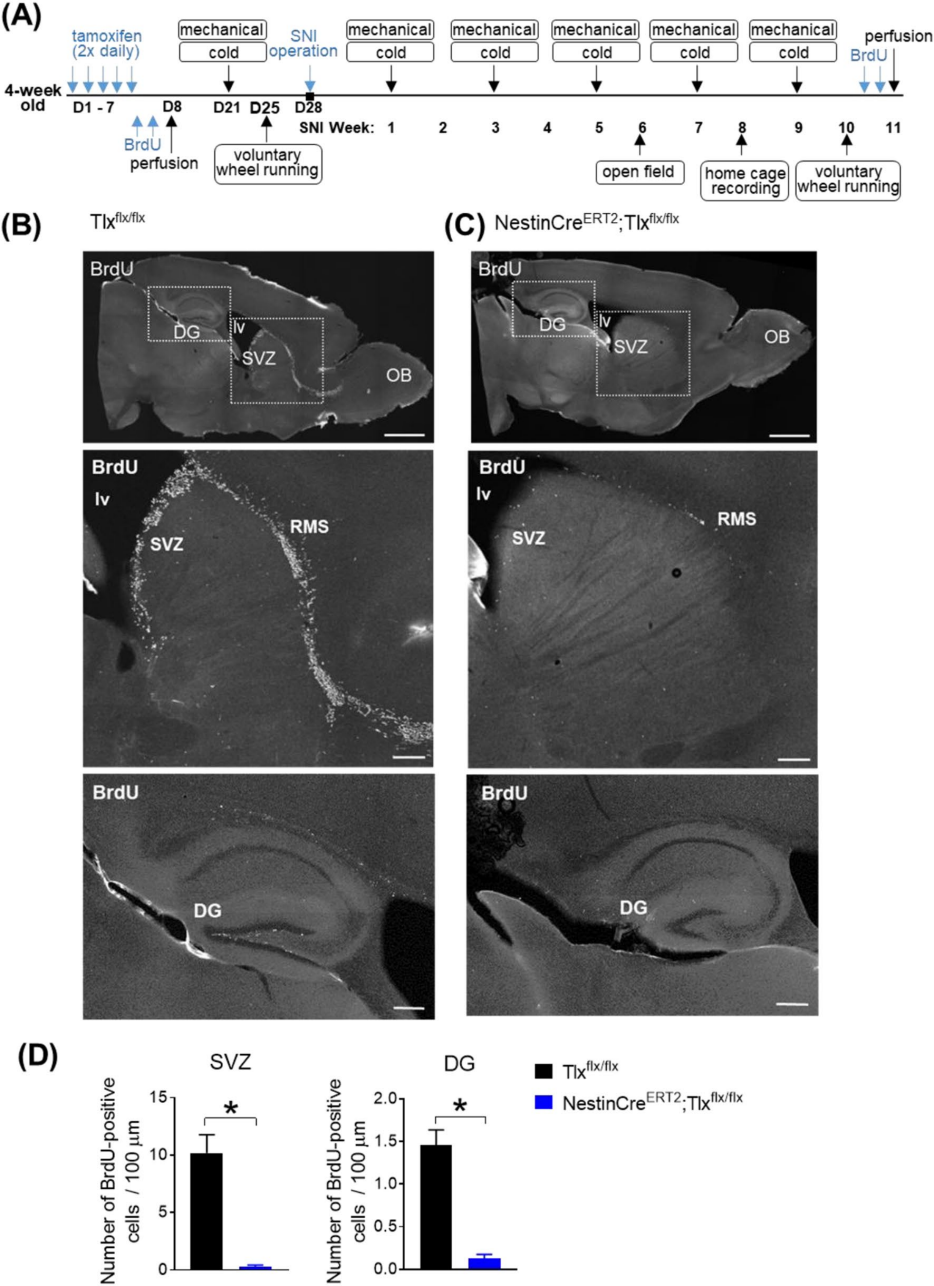
(**A**) Scheme of experimental timeline of injections, nerve operation and behavioural assessments carried out over the course of 15 weeks. (**B–D**) On experimental Day 8, neurogenesis (indicated by BrdU-labelled cells) in the SVZ and DG of NestinCre^ERT2^;TIx^flx/flx^ mice is abolished after Tamoxifen injections (twice daily over 5 consecutive days) followed by BrdU administration (once daily for 2 days after Tamoxifen administration to label proliferating cells). (**B,C**) Example images of sagittal whole brain sections (upper row; scale bar, 1 mm) with BrdU-labelled cells (in white) present along the rostral migratory stream (RMS) of control cre-negative TIx^flx/flx^ mice (in (**B**)) after Tamoxifen injections. BrdU-labelled cells are largely absent along the RMS of cre-positive NestinCre^ERT2^;TIx^flx/flx^ mice (in (**C**)) after Tamoxifen injections. Higher magnification of regions indicated in the dotted box are shown in the lower panels (scale bar, 200 pm). (**D**) Average number of BrdU-positive cells (per 100 pm) present in the SVZ (left) and DG (right) of either cre-negative or cre-positive NestinCre^ERT2^;TIx^flx/flx^ mice, 1 week after Tamoxifen injection (experimental Day 8; *n* = 5–6/group; *p < 0.05, unpaired I-test). *OB* olfactory bulb, *lv* lateral ventricle, *DG* dentate gyrus, *SVZ* subventricular zone.

All animal experimental procedures in this study were performed in accordance to the ethical guidelines set by the local governing body (Regierungspräsidium Karlsruhe, Germany; approval number 35-9185.81/G-199/14) and the German Cancer Research Institute (DKFZ) Animal Care guidelines. All experiments were carried out in compliance with ARRIVE guidelines on animals. Adult male (4 weeks old) NestinCre^ERT2^/Tlx floxed mice^13^ were used and were housed in standard housing conditions in a 12 h light/dark cycle with access to water and food ad libitum. Cre^ERT2^ activation was carried out with intraperitoneal (i.p.) injections of 1 mg/day tamoxifen (Sigma-Aldrich; dissolved in ethanol (65 mg/ml) and diluted in corn oil to a final concentration of 10 mg/ml) twice a day for 5 consecutive days in 4 week old animals (Days 1 to 5; referred to as D1–5 in experimental timeline shown in Fig. 1A).

### Bromodeoxyuridine (BrdU) injections

To label proliferating cells in cre-negative control Tlx floxed mice and cre-positive NestinCre^ERT2^/Tlx floxed mice, BrdU (50 mg/kg, Sigma Aldrich catalog #B5002, dissolved in NaCl solution) was administered i.p*.* daily for 2 days (Days 6 to 7; referred to as D6-7 in experimental timeline shown in Fig. 1A). The animals were killed 24 h after the last BrdU administration. An initial group of cre-negative control Tlx floxed mice and cre-positive NestinCre^ERT2^/Tlx floxed mice were perfused on day 8 (referred to as D8 in experimental timeline shown in Fig. 1A) to confirm for loss of proliferating cells in the cre-positive animals before any behavioural assessments and nerve injury was carried out. Additionally, BrdU was also administered i.p. daily for 2 days 11 weeks post-SNI (24 h before perfusion, Fig. 1A).

### Neuropathic pain model—spared nerve injury (SNI)

Three weeks after the end of tamoxifen treatment, the animals were placed under anesthesia with 2% isoflurane and an incision (~ 2 cm) was made to the lateral skin surface and biceps femoris muscles of the right thigh to expose the sciatic nerve and its branches (sural, common peroneal and tibial nerves). The common peroneal and tibial nerves were tightly ligated with a silk suture and a section of the nerve bundle (2–4 mm) was cut and removed distal to the ligation, leaving the sural nerve intact. The skin was subsequently sutured close and animals left to recover in a heated cage for 24 h. Behavioral testing was carried out 7 days post-operation.

### Behavioural tests

Behavioural experiments were performed 2 weeks after the end of the tamoxifen injections (pre-SNI) and starting from 1 week after SNI (SNI Week 1 onwards; experimental timeline in Fig. 1A). All behavioral measurements were carried out during the light cycle of the animals. The experimenter was blinded to the identity of the mice throughout the experiments.

#### von Frey test

Animals were acclimatized to the behavioral setup with two 45 min sessions prior to the testing day. Measurement of the mechanical sensitivity of hind paws was carried out via applications of von Frey filaments with increasing forces (0.04 to 1.4 g) to the lateral (SNI animals) surfaces of the hind paws and withdrawal frequencies were recorded (5 applications per filament applied at 1 min intervals). The 40% mechanical thresholds were determined by the filament force which elicited ≥ 40% withdrawal.

#### Cold plate test

The paw withdrawal latency to 2 °C was assessed with the cold plate test (Bioseb, France). A cut-off threshold of 30 s was used to prevent potential tissue damage to the paw surfaces.

#### Open field exploratory test

The open field test was performed with a 44 × 44 cm box under dim light conditions (40–60 lx). Each mouse was placed in the center of the box and its exploration activity was recorded for 10 min via the ANY-maze tracking software (Stoelting Co., Ireland). A middle square comprising of 13 × 13 cm was designated as the center zone and the surrounding areas used as the margin zone. A 3 cm border along the walls of the box was designated as the thigmotaxis zone. Parameters indicative of locomotion (e.g. total distance ran, mean speed), fear-like (e.g. freezing) and anxiety-like (e.g. time spent in the center or thigmotaxis zones) behaviours were analyzed.

#### Spontaneous wheel running

The voluntary wheel running behavior was assessed with the activity wheel system (Lafayette Instrument, USA). Briefly, animals were placed individually for 24 h in cages fitted with an electronic activity wheel. The total distance of running (measured by number of wheel turns) made by the animal was monitored in real-time and represented as an accumulative running distance recorded per hour over 24 h (light cycle: 07:00 to 19:00; dark cycle: 19:00 to 07:00).

### Immunohistochemisty

Mice were deeply anesthetized and transcardially perfused with 4% paraformaldehyde (PFA). The brains were collected, further post-fixed in 4% PFA overnight at 4 °C and cut with a vibratome (50 µm thick free-floating sections; Leica VT100S, Leica Microsystems GmbH). For BrdU stainings, the sections were incubated in 1 M HCl at 45 °C for 45 min and subsequently neutralized with 10 mM Tris (pH 8.5) at room temperature before permeabilization and blocking. Sections were permeabilized in 0.2% triton in phosphate-buffered saline (PBS) for 30 min and blocked in 3% bovine serum albumin in PBS (BSA-PBS) for 30 min before incubation with the primary antibody in 3% BSA-PBS overnight at 4 °C. The primary antibodies used were: anti-BrdU (mouse; BD Biosciences, catalog 47580; 1:1000), anti-DCX (goat; Santa Cruz, catalog sc-8066; 1:500) and anti-Ki67 (rabbit; Abcam, catalog ab15580; 1:250). The sections were washed three times in PBS and further incubated with secondary antibodies in 3% BSA-PBS for 1 h at room temperature followed by a PBS wash and mounted. The secondary antibodies used were: donkey anti-rabbit Cy3 (Jackson ImmunoResearch catalog #711-165-152, donky anti-goat Alexa 647 (Invitrogen catalog #A-21447), donkey anti-mouse Alexa488 (Invitrogen catalog #A21202). DAPI (Invitrogen catalog #D1306; 1:100; 5 min) was used to stain the nuclei.

### Image acquisition

**Figure 2:**
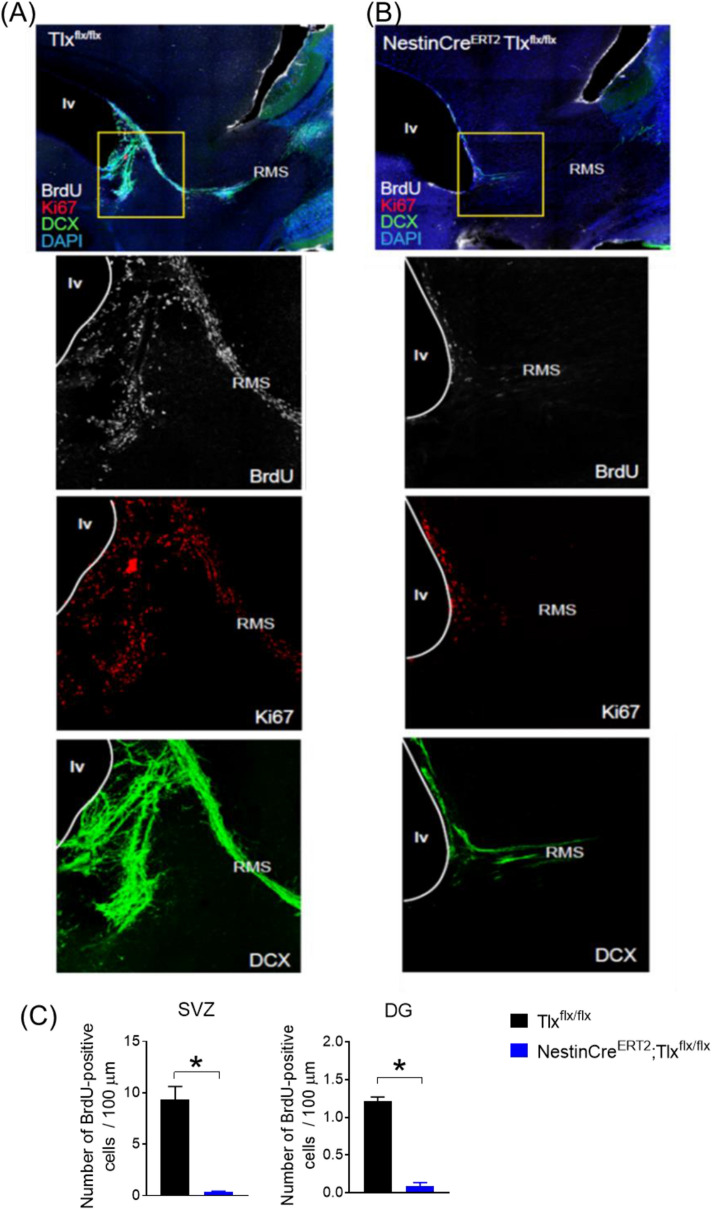
Assessment of newborn cells in the RMS 10 weeks after SNI in control TIx^flx/flx^ (**A**) and NestinCre^ERT2^;TIx^flx/flx^ (**B**) mice. Representative immunofluorescent images of the SVZ and RMS (first row) and high magnification of boxed region (lower panels) displaying BrdU-, Ki67- and DCX-labelled cells. A major absence of neurogenesis is still observed in the NestinCre^ERT2^;TIx^flx/flx^ mice 15 weeks after tamoxifen injections. (**C**) Average number of BrdU-positive cells (per 100 pm) present in the SVZ (left) and DG (right) of either cre-negative or cre-positive NestinCre^ERT2^; TIx^flx/flx^ mice, 15 weeks after Tamoxifen administration (SNI week 11) (*n* = 2–3/group; *p < 0.05, unpaired I-test). *lv* lateral ventricle, *RMS* rostral migratory stream, *DG* dentate gyrus, *SVZ* subventricular zone.

Images were acquired with a LSM 700 Zeiss laser-scanning confocal microscope to visualize the immunofluorescence levels of the stained sections. Z-stack images were maximally projected using the ImageJ software (version 1.53c, National Institutes of Health, USA) and overlaid with the corresponding atlas^14^ section to anatomically identify the regions of interest depicted in Figs. 1 and 2.

### Statistical analysis

Data are presented as mean ± standard error of the mean (S.E.M) and analyzed using GraphPad Prism (version 7.05). Data from behavioral experiments were examined either using the one-way ANOVA repeated measures with Dunnett’s, one-way ANOVA with Dunn’s or two-way ANOVA repeated measures with Tukey’s multiple comparison tests as indicated in legend text. Total number of BrdU cells were counted in z-stack projection images from 50 μm sagittal brain sections and were normalized either to the length of the lateral ventricle (SVZ results) or the DG. BrdU cell counts were examined using the unpaired Student’s *t*-test. A *p* value of < 0.05 was considered significant in all tests.

## Results

### Inducible knockout of Tlx permanently abolishes neurogenesis in the adult mouse brain

In order to investigate the functional changes in sensory, emotional and ambulatory behaviours due to an in vivo loss of neurogenesis in the adult mouse brain, we employed a mouse line wherein an irreversible knockout of *Tlx* (Tailless) is driven by a Tamoxifen-inducible Cre recombinase expressed under the Nestin promotor (NestinCre^ERT2^;Tlx^flx/flx^)^15^. *Tlx* encodes an orphan nuclear receptor of the nuclear receptor subfamily 2 group E (NR2E) subclass that is specifically expressed in neural stem cells of the two largest germinal neurogenesis zones in the adult brain, namely the subventricular zone (SVZ) of the lateral ventricle where new neurons are generated and then migrate through the rostral migatory stream (RMS) to the olfactory bulb, and the dentate gyrus (DG) of the hippocampus^16^. *Tlx* predominant functions as a transcriptional repressor^17^ and its expression has been shown to be critically important in the proliferation, growth and patterning of neural stem cells, and genetic deletion of *Tlx* leads to a complete loss of neurogenesis^13,18,19^. At adult stages, Tlx is highly expressed particularly in the SVZ^13^ and co-express Nestin, a neural stem cell marker and filament protein^20^.

To inactivate Tlx and abolish neurogenesis, we injected Tamoxifen (twice daily over 5 consecutive days) in 4-weeks-old mice followed by BrdU administration (once daily for 2 days after Tamoxifen administration) to label proliferating cells. To confirm the loss of proliferating cells, animals were perfused one day post-BrdU treatment (Day 8 in experimental timeline shown in Fig. 1A). A major loss of BrdU-labelled cells in the SVZ and RMS was observed in the cre-positive NestinCre^ERT2^;Tlx^flx/flx^ mice compared to the cre-negative control Tlx^flx/flx^ littermates before the start of behavioural assessments and induction of nerve injury (Fig. 1B,C). This was additionally reflected in a significantly lower number of BrdU-positive cells observed in the SVZ (control: 10.2 ± 1.6 *vs* cre-positive: 0.3 ± 0.1, *p* < 0.0001) and DG (control: 1.5 ± 0.2 *vs* cre-positive: 0.1 ± 0.05, *p* = 0.0004; Fig. 1D).

To confirm that the Tamoxifen-induced deletion of *Tlx* was long-lasting and that loss of neurogenesis was still detected 11 weeks after nerve injury, we injected BrdU (once daily for 2 days) 24 h before perfusing the animals. In order to label newborn proliferating cells, we stained the brain tissue with BrdU, Ki67 as well as a marker of neuronal precursor cells, namely doublecortin (DCX). Indeed, examination of the RMS and lateral ventricular regions indicated that proliferating neuronal precursor cells remained largely absent in the NestinCre^ERT2^;Tlx^flx/flx^ mice, in contrast to control mice (Fig. 2A,B). This was also observed by a significantly lower number of BrdU-positive cells in the SVZ (control: 9.3 ± 1. 3 *vs* cre-positive: 0.4 ± 0.03, *p* = 0.0022) and DG (control: 1.2 ± 0.05 *vs* cre-positive: 0.09 ± 0.05, *p* = 0.0006; Fig. 2C). No significant differences were observed in the number of BrdU-positive cells in the SVZ and DG of the control versus the cre-positive animals on experimental Day 8 compared to week 15 (control SVZ: 10.2 ± 1.6 *vs* 9.3 ± 1. 3, *p* > 0.05; cre-positive SVZ: 0.3 ± 0.1 *vs* 0.4 ± 0.03, *p* > 0.05; control DG: 1.5 ± 0.2 *vs* 1.2 ± 0.05, *p* > 0.05; cre-positive DG: 0.1 ± 0.05 *vs* 0.09 ± 0.05, *p* > 0.05).

### Loss of adult neurogenesis dampens the development and accelerates the recovery of chronic mechanical allodynia induced by neuropathic injury

To examine the in vivo functional effects of attenuated neurogenesis on pathological pain, we employed the SNI model to induce chronic neuropathic pain over a course of 9 weeks in cre-positive NestinCre^ERT2^;Tlx^flx/flx^ and cre-negative control Tlx^flx/flx^ mice that received Tamoxifen administration 4 weeks prior to the start of the behavioral measurements (when animals were 8 weeks old). Mechanical sensitivity of the hind paw was assessed with plantar applications of graded forces using von Frey filaments (0.04–1.4 g) pre-SNI operation as well as 1, 3, 5, 7, and 9 weeks post-SNI operation (full experimental timeline shown in Fig. 1A).

**Figure 3:**
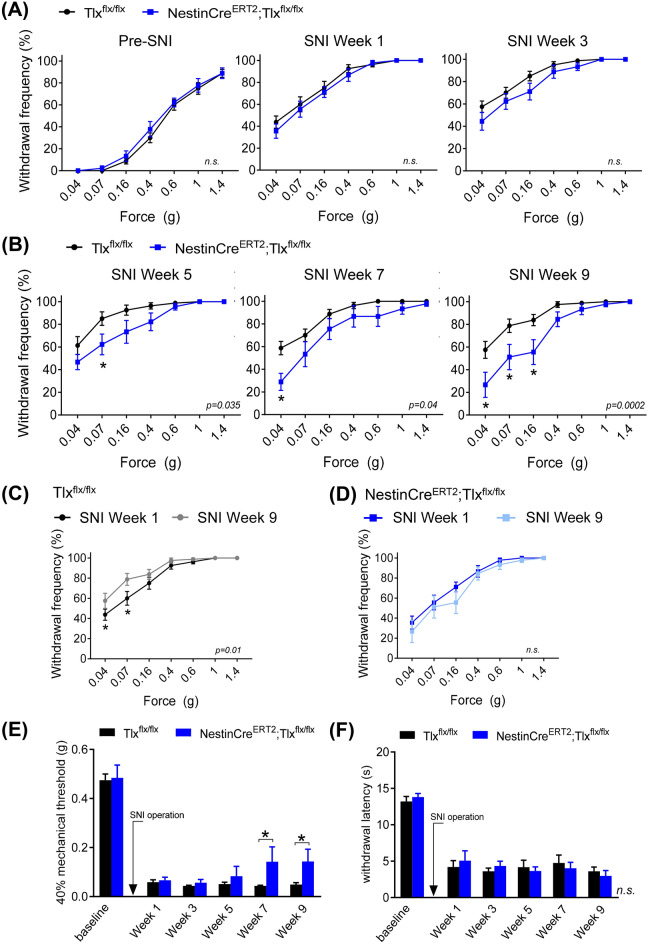
(**A,B**) Withdrawal frequency of hind paw to applications of graded von Frey forces pre-injury and 1, 3, 5, 7 and 9 weeks after sciatic nerve injury (SNI) in control TIx^flx/flx^ and NestinCre^ERT2^;TIx^flx/flx^ mice (*n* = 16 and 9, respectively). (**C,D**) Comparison of paw withdrawal frequency to von Frey forces at early (week 1) versus late (week 9) stages after SNI in control TIx^flx/flx^ mice (**C**) and NestinCre^ERT2^;TIx^flx/flx^ mice (**D**). (**E**) Mechanical threshold and (**F**) withdrawal latency to cold (2 °C) recorded pre-injury and at 1, 3, 5, 7 and 9 weeks after SNI operation in control TIx^flx/flx^ and NestinCre^ERT2^;TIx^flx/flx^ mice. *p* values in figure panels indicate significance between entire curves. *p < 0.05, two-way ANOVA (**A,B**) with repeated measures (**C–F**); *n.s.* not significant.

We observed that pre-SNI surgery, basal mechanical sensitivity of the hind paw are comparable between NestinCre^ERT2^;Tlx^flx/flx^ and Tlx^flx/flx^ control mice (n = 9 and 16, respectively). Here, withdrawal frequencies to all forces applied were not significantly different between the two groups at baseline (left panel in Fig. 3A). After SNI operation, both groups of mice developed a similar extent of mechanical hypersensitivity in the injured paw, observed by an increase in withdrawal frequencies to all forces applied, that was observed up to 3 weeks after the surgery (middle and right panels in Fig. 3A). Interestingly, at later states of chronic neuropathy (weeks 5, 7, and 9 post-SNI), we observed a significant attenuation of SNI-induced mechanical hypersensitivity in the NestinCre^ERT2^;Tlx^flx/flx^ mice compared to the Tlx^flx/flx^ control mice, which became more profound over time (week 5: *p* = 0.035 vs week 9: *p* = 0.0002; Fig. 3B). At 9 weeks post-SNI, paw withdrawal frequencies to low mechanical forces were significantly lower in the NestinCre^ERT2^;Tlx^flx/flx^ mice compared to the Tlx^flx/flx^ control mice (NestinCre^ERT2^;Tlx^flx/flx^ *vs* Tlx^flx/flx^, 0.04 g: 27 ± 11 *vs* 58 ± 8%, *p* = 0.0014; 0.07 g: 51 ± 11 *vs* 79 ± 6%, *p* = 0.0057; 0.16 g: 56 ± 11 *vs* 84 ± 5%, *p* = 0.0045; Fig. 3B).

Furthermore, when we compared the extent of mechanical hypersensitivity developed 9 weeks versus 1 week after SNI, we observed that the Tlx^flx/flx^ control mice became significantly more hypersensitive at later stages of SNI (*p* = 0.01, Fig. 3C). The NestinCre^ERT2^;Tlx^flx/flx^ mice, however, did not further develop a more severe hypersensitive phenotype after 1 week post-SNI operation (*p* = 0.08, Fig. 3D).

When we analysed the 40% mechanical thresholds, we also observed that at later SNI stages (weeks 7 and 9 post-operation), there was a significant increase in the paw mechanical thresholds of the NestinCre^ERT2^;Tlx^flx/flx^ mice compared to the Tlx^flx/flx^ control mice (NestinCre^ERT2^;Tlx^flx/flx^ *vs* Tlx^flx/flx^, week 7: 0.14 ± 0.06 *vs* 0.04 ± 0.003 g, *p* = 0.034; week 9: 0.14 ± 0.05 *vs* 0.05 ± 0.008 g, *p* = 0.049; Fig. 3E), suggesting an onset of recovery of SNI-induced mechanical allodynia 7 weeks after nerve injury in the NestinCre^ERT2^;Tlx^flx/flx^ mice. Interestingly, we observed no differences in the cold sensitivity (assessed by paw withdrawal latency to 2 °C) between both groups at all the time points examined (Fig. 3F). These results reveal that specifically the late, chronic stages of SNI-induced mechanical hypersensitivity are dependent on adult neurogenesis.

### Attenuation of SNI-induced chronic mechanical hypersensitivity in NestinCre^ERT2^;Tlx^flx/flx^ mice is not due to changes in locomotory function

**Figure 4:**
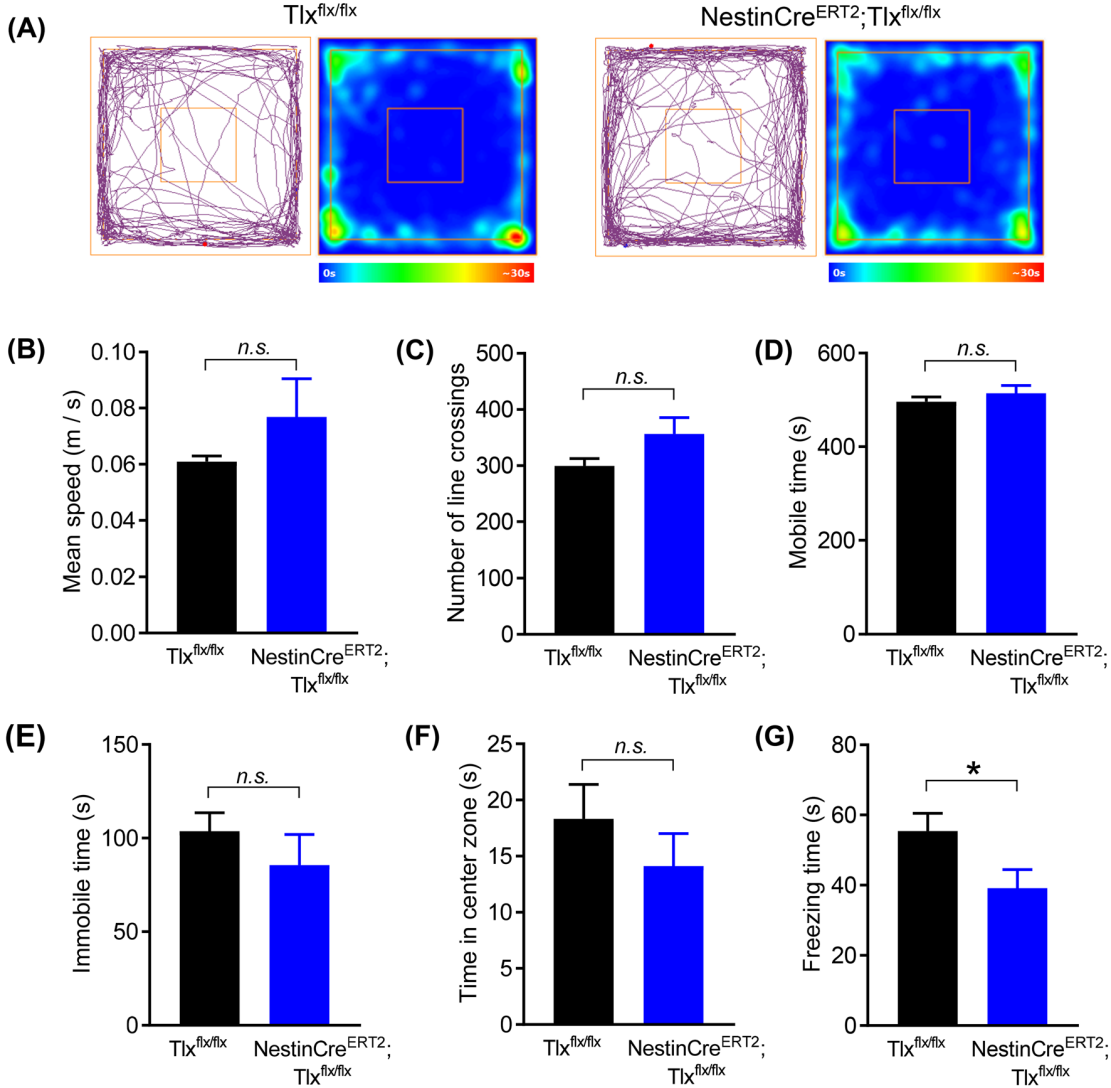
(**A**) Example track plots and heat time maps of exploratory behaviour and locomotion in the open field test at late stage SNI. (**B–G**) Locomotive behaviors assessed from the open field test—mean speed (**B**), number of line crossings (**C**), time spent mobile (**D**), time spent immobile (**E**), time spent in the open center zone (**F**) and total freezing time (**G**). *p < 0.05, unpaired t-test (**B–G**); *n.s.* not significant.

To determine if the changes in SNI-induced mechanical hypersensitivity (i.e. paw withdrawal) in the NestinCre^ERT2^;Tlx^flx/flx^ mice was due to changes in locomotory performance, we performed the novel exploratory open field test at late stage SNI (6 weeks post-operation). Here, we observed no differences in the motor activity of both the NestinCre^ERT2^;Tlx^flx/flx^ and Tlx^flx/flx^ control mice (tracking plot examples in Fig. 4A). Analysis of the mean speed, time spent mobile and the total number of line crossings recorded during the test did not differ between both groups (Fig. 4B–G). Although the NestinCre^ERT2^;Tlx^flx/flx^ mice showed a trend for increased ambulatory behaviour, these did not reach significance (NestinCre^ERT2^;Tlx^flx/flx^ *vs* Tlx^flx/flx^, mean speed: 0.077 ± 0.016 *vs* 0.061 ± 0.06 m/s, *p* = 0.14 in Fig. 4B; line crossings: 357 ± 29 *vs* 300 ± 13, *p* = 0.05 in Fig. 4C). Both groups spent comparable amount of time being mobile and immobile during the test (NestinCre^ERT2^;Tlx^flx/flx^ *vs* Tlx^flx/flx^, mobile: 514 ± 16 *vs* 496 ± 10 s, *p* = 0.33 in Fig. 4D; immobile: 86 ± 16 *vs* 104 ± 9 s, *p* = 0.33 in Fig. 4E). Furthermore, no differences in the amount of time spent exploring the open center arena were observed, suggesting that anxiety-like states were similar between the neuropathic NestinCre^ERT2^;Tlx^flx/flx^ and control Tlx^flx/flx^ mice (14.11 ± 2.91 *vs* 18.33 ± 3.05 s, *p* = 0.37 in Fig. 4F). Interestingly, the total freezing time exhibited were significantly reduced in the NestinCre^ERT2^;Tlx^flx/flx^ mice compared to the control group, indicating a state of reduced fear-like levels in these mice lacking newborn neurons (39.13 ± 5.32 *vs* 55.44 ± 5.05 s, *p* = 0.049 in Fig. 4G).

### Loss of neurogenesis in neuropathic NestinCre^ERT2^;Tlx^flx/flx^ mice is also accompanied by an increase in home cage activity and an improved state of well-being

**Figure 5:**
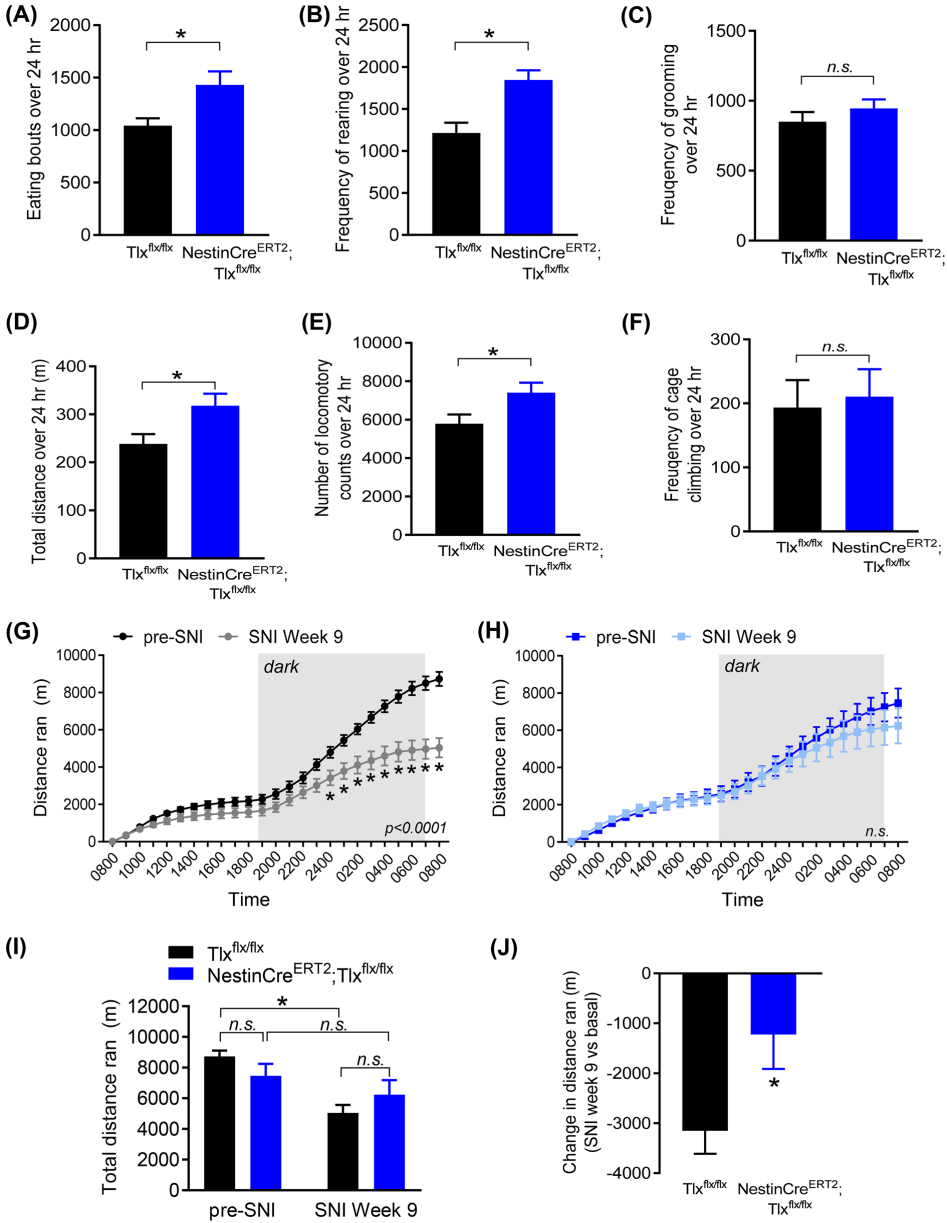
Assessment of spontaneous and voluntary behaviors. (**A–F**) LABORAS home cage behavioural recording over 24 h of eating bouts (**A**), rearing (**B**) and grooming (**C**) frequencies as well as total movement (**D**), locomotory counts (**E**) and frequency of cage climbing (**F**) 8 weeks after SNI operation in control TIx^flx/flx^ and NestinCre^ERT2^; TIx^flx/flx^ mice. (**G,F**) Comparison of the 24 h voluntary wheel running distances pre- and post-SNI in the control TIx^flx/flx^ (**G**) and NestinCre^ERT2^;TIx^flx/flx^ mice (**H**). (**I**) Comparison of the total distance ran over 24 h in the wheel and (**J**) comparison of the change in total distance ran in the wheel 9 weeks post-SNI versus the respective pre-injury values in individual animal. *p < 0.05, unpaired t-test (**A–F,J**), two-way ANOVA with repeated measures (**G–I**); *n.s.* not significant.

In addition to diminished neuropathic mechanical hypersensitivity, NestinCre^ERT2^;Tlx^flx/flx^ mice also displayed significantly higher home cage ambulatory movements and improved appetite compared to the control Tlx^flx/flx^ mice at 8 weeks post-SNI operation. Automatic tracking recording of individual mouse over a 24 h period indicated that neuropathic NestinCre^ERT2^;Tlx^flx/flx^ mice displayed significantly more eating bouts (1429 ± 130 *vs* 1042 ± 70, *p* = 0.009; Fig. 5A) and rearing behaviours as compared to the control group (1845 ± 117 *vs* 1214 ± 122, *p* = 0.003; Fig. 5B), although grooming counts remained comparable between the two groups (944 ± 65 *vs* 850 ± 70, *p* = 0.38; Fig. 5C). Because behaviors were not compared prior to SNI, it cannot be ruled out that these changes are owing to Tlx deletion independently of neuropathy.

Furthermore, the NestinCre^ERT2^;Tlx^flx/flx^ mice with SNI exhibited significantly higher periods of active movements compared to the floxed control mice with SNI (total movement: 317.4 ± 25.9 *vs* 238.3 ± 20.5 m, *p* = 0.027 in Fig. 5D; locomotory counts: 7398 ± 534 *vs* 5782 ± 486, *p* = 0.045 in Fig. 5E). The frequency of cage climbing, however, was unaffected (210 ± 43 *vs* 193 ± 43, *p* = 0.80; Fig. 5F). These results suggest a higher level spontaneous locomotive activity in the neuropathic animals lacking adult neurogenesis, which can be either interpreted as an indicator of improved motivation, thus suggesting overall well-being, or can be purely a consequence of reduced ongoing pain and mechanical allodynia.

Voluntary wheel running in neuropathic animal models have only recently been used as a non-evoked measure to access the animal’s well-being and motivation as well as a potential readout for spontaneous ongoing pain in injured animals^21,22^. Here, we observed that 9 weeks after SNI, control Tlx^flx/flx^ mice ran significantly less over a 24 h cumulative period (12 h light/12 h dark) compared to pre-injury (*p* < 0.0001, Fig. 5G), particularly during the dark (i.e. active) period. However, NestinCre^ERT2^;Tlx^flx/flx^ animals showed no difference in their 24 h running profile 9 weeks after SNI compared to pre-injury (*p* = 0.4, Fig. 5H). When we compared the total distance ran between the two groups, we noted that although there were no differences observed between the two groups pre- and post-injury, the control Tlx^flx/flx^ mice ran significantly less after injury (pre-SNI: 8729 ± 377, SNI Week 9: 5039 ± 519 m, *p* < 0.0001; Fig. 5I), which was also reflected in the change in total distance ran compared to pre-injury conditions (NestinCre^ERT2^;Tlx^flx/flx^ *vs* Tlx^flx/flx^: − 1223 ± 689 *vs* − 3149 ± 462 m, *p* = 0.02; Fig. 5J), supporting the above observations that mice lacking neurogenesis either have a better state of motivation and well-being or reduced ongoing pain, or both during chronic neuropathy.

Taken together, our results suggest that ongoing neurogenesis in the adult brain contributes to the maintenance of mechanical hypersensitivity and comorbid symptoms of neuropathic pain, particularly over late, chronic phases of the pain disorder.

## Discussion

The results from our study show that adult neurogenesis is involved in the maintenance of pathological pain induced by peripheral neuropathy. Using inducible transgenic NestinCre^ERT2^;Tlx^flx/flx^ mice, we demonstrate that abolishing adult neurogenesis before the onset of injury had no effects on the emergence of hypersensitivity-associated behaviors, but instead promoted the recovery of tactile allodynia at chronic stages post-injury, which was independent of motor function. Furthermore, attenuation adult neurogenesis enhanced the state of well-being and spontaneous, voluntary activity in these nerve-injured animals.

Here, we used an inducible means to irreversibly abolish neurogenesis in the adult mice immediately prior to nerve injury, in order to determine the functional role of newly generated cells during the development and maintenance phases of pathological pain. The absence of newborn proliferating cells was confirmed before the onset of injury and the loss of neurogenesis persisted for up to 15 weeks throughout the behavioural assessments. Importantly, the genetic engineering tool employed here is highly specific for abolishing adult neurogenesis in the brain, in contrast to pharmacological reagents inhibiting proliferation that do not differentiate between newly generated neurons, glia and other proliferating cells. This aspect, coupled with the inducible nature of the manipulation, allowed for a selective ablation of adult neurogenesis for the investigation of neuropathic pain and comorbid affective behaviors in this study.

In this study, we show that neurogenesis-mediated mechanisms do not appear to be involved in the developmental early stages of pain onset, but rather becomes a major contributor at later stages of pain chronicity. Indeed, the absence of adult neurogenesis likely prevented the maladaptive processes in mesolimbic circuits, where we have recently shown that a proportion of adult neural stem cells in the SVZ have the capacity to integrate into pre-existing circuits and mature into medium spiny projection neurons in the nucleus accumbens^12^. Intriguingly, newly generated neurons in the nucleus accumbens are significantly increased 6 weeks after sciatic nerve injury^12^ and our current result demonstrates that the absence of adult neurogenesis significantly attenuated painful behaviors after 5 weeks post-nerve injury. Taken together, given the similar time course, we speculate that the functional integration of newly generated medium spiny neurons into pre-existing circuits within the nucleus accumbens subserve a pro-nociceptive role. Such a candidate mechanism would potentially facilitate the maintenance of pathological pain. How these specific new neurons functionally interact with local mesolimbic circuits during pain chronicity will need to be further explored in future studies.

Although the genetic mouse model we used precludes us from determining which specific neurogenic areas of the brain are involved in the chronic pain phenotypes observed in this study, available evidence suggests that newborn cells maturing and integrating in regions such as the hippocampus, amygdala, nucleus accumbens and prefrontal cortical regions likely orchestrate a cascade of plastic changes at the cellular and circuit level over the course of months after injury^1,5,12,23^. Such reorganisation of network activity in these brain regions has been shown to underlie the altered sensorimotor and emotional-motivational processing that are frequently observed in chronic pain patients and preclinical models^24–26^. Importantly, motivational processes influencing pain aversiveness and reward from pain relief are highly encoded by mesocorticolimbic circuitries^26,27^ that commonly engage the nucleus accumbens, amygdala, anterior cingulate and prefrontal cortices, regions where a proportion of newly generated cells are known to migrate to and populate. In humans, a facilitatory role for prefrontal-nucleus accumbens connectivity has been positively correlated with pain persistence in chronic back pain patients^28,29^ while in preclinical models of chronic pain, selective optogenetic manipulation of projections to the nucleus accumbens also significantly impacts chronic pain and associated negative affective behaviors^30,31^.

Within the medial shell of the nucleus accumbens, activity of the GABAergic indirect spinal project neurons (iSPN) that reside in this region has been associated with aversiveness and negative affect. For instance, increases in excitability and altered morphology of iSPNs are typically observed after a peripheral nerve injury and dampening activity or inactivation of these neurons diminishes injury-induced tactile allodynia^32^. This suggests that the absence of newly integrated neurons may prevent or diminish the maladaptive excitability and consequent structural changes in pre-existing iSPNs in the nucleus accumbens, which could underlie the reduced allodynia and ongoing pain as well as an enhanced state of well-being and motivation (for voluntary running) observed in our injured animals lacking adult neurogenesis. It is important to bear in mind, however, that changes in some of these behaviors, such as voluntary running and locomotion, may also be an indirect consequence of reduced mechanical hypersensitivity observed in mice lacking adult neurogenesis in late stages of neuropathy. It is also possible that the lack of new neurons entering other cortical and subcortical regions contributed to the improved appetite and reduced fear-like state we observed in this study. For example, distinct populations of central and basolateral amygdala neurons, areas where neurogenic precursor cells also exists^1,5^, have been recently shown to regulate appetitive behaviors^33^ while projections between the basolateral amygdala, prefrontal and anterior cingulate areas have been well-described to modulate innate fear responses and memory^34,35^. Again, a caveat that should be considered is that Tlx deletion may alter food intake independently of neuropathy, which needs to be tested in future studies.

Thus far, the role of cortical neurogenesis in pain has remained unclear. Stimulation of cortical neurogenesis is highly influenced by ischemic insults and inflammation^36,37^ that lead to disturbances in spontaneous cortical network activity. However, despite the major roles of the cortex in sensory and cognitive processes underlying pain, it is surprising that the impact of cortical neurogenesis on chronic pain is now only beginning to be explored. Interestingly, some of these newly generated neurons, for example, those in the motor cortex, have been suggested to be capable of developing projections into the spinal cord^38^. Such an observation raises the possibility that dysregulation of adult cortical newborn neurons could arise from painful injuries and thus influences spinal sensory and nociceptive transmission through cortico-spinal projections^39,40^. Furthermore, such altered maturation and integration of new neurons in the cortex may lead to functional impairments in synaptic connectivity and network activity, which would have consequences on distinct cortical oscillatory patterns described in regions such as the prefrontal and primary somatosensory cortices during pathological pain^41–43^.

In addition, the results of our study extends previous reports that implicate adult hippocampal neurogenesis in both neuropathic and inflammatory persistent pain states^23,44^. For instance, using a multi-array of tools including pharmacological intervention with antimitotic AraC, x-irradiation ablation and transgenic animals to functionally downregulate the levels of adult neurogenesis in the hippocampus blocked persistent pain; conversely, upregulating neurogenesis extended painful behaviors in nerve-injured mice^23^. However, partial pharmacological blockade of cell division specifically in the hippocampus successfully reduced nerve injury-induced mechanical hypersensitivity at an earlier time point (already at 2 weeks after nerve injury), suggesting that active hippocampal proliferating cells are associated with the acquisition of persistent pain behavior^23^. On the other hand, our current animals lacking global newborn neurons developed a normal extent of tactile allodynia after nerve injury. The difference in observations suggests that the disruption of new glial proliferation, rather than neurons, may be the dominant contributor to the emergence of persistent pain during the early phases post-injury observed in their study, while newborn neurons, which usually take weeks to integrate into functional circuits, have a more evident role in maintaining post-injury pain at chronic stages as we have observed here. Indeed, in animals where they employed long-term ablation of adult hippocampal neurogenesis with x-irradiation, a reversal of tactile allodynia several weeks after nerve injury was observed, similar to our current findings. On the other hand, since proliferation of adult neural stem cells was abolished across the dentate gyrus and SVZ in our study, it was surprising to detect no changes in the development of pain phenotype after injury. Future studies will need to be carried out to clarify if ongoing adult neurogenesis in specific brain regions have differential properties during the course of pathological pain.

Altogether, our study provides direct and specific evidence for the importance of adult neurogenesis in the maintenance of long-lasting neuropathic chronic pain as well as in the debilitating reduction of quality of life parameters. Since patients often seek medical assistance when pain is chronic and a majority of clinical cases that are therapy-resistent involve chronic stages of neuropathic pain, our results hold significant therapeutic relevance. Elucidating the mechanisms and molecular triggers influencing the regulation of adult neurogenesis post-injury could additionally reveal potential therapeutic targets that may aid in accelerating recovery in chronic pain patients and improving their quality of life.
